# An Optimization Method for Non-IID Federated Learning Based on Deep Reinforcement Learning

**DOI:** 10.3390/s23229226

**Published:** 2023-11-16

**Authors:** Xutao Meng, Yong Li, Jianchao Lu, Xianglin Ren

**Affiliations:** 1 School of Computer Science and Engineering, Changchun University of Technology, Changchun 130012, China; mengxutao1103@gmail.com (X.M.); 2202103094@stu.ccut.edu.cn (X.R.); 2 AI Research Institute, Changchun University of Technology, Changchun 130012, China; 3 School of Computer Science and Technology, Jilin University, Changchun 130012, China; 4 School of Computing, Macquarie University, Sydney, NSW 2109, Australia; jianchao.lu@hdr.mq.edu.au

**Keywords:** federated learning, deep reinforcement learning, non-IID, client selection

## Abstract

Federated learning (FL) is a distributed machine learning paradigm that enables a large number of clients to collaboratively train models without sharing data. However, when the private dataset between clients is not independent and identically distributed (non-IID), the local training objective is inconsistent with the global training objective, which possibly causes the convergence speed of FL to slow down, or even not converge. In this paper, we design a novel FL framework based on deep reinforcement learning (DRL), named FedRLCS. In FedRLCS, we primarily improved the greedy strategy and action space of the double DQN (DDQN) algorithm, enabling the server to select the optimal subset of clients from a non-IID dataset to participate in training, thereby accelerating model convergence and reaching the target accuracy in fewer communication epochs. In simulation experiments, we partition multiple datasets with different strategies to simulate non-IID on local clients. We adopt four models (LeNet-5, MobileNetV2, ResNet-18, ResNet-34) on the four datasets (CIFAR-10, CIFAR-100, NICO, Tiny ImageNet), respectively, and conduct comparative experiments with five state-of-the-art non-IID FL methods. Experimental results show that FedRLCS reduces the number of communication rounds required by 10–70% with the same target accuracy without increasing the computation and storage costs for all clients.

## 1. Introduction

The application of deep learning technology in the Internet of Things (IoT) is very common, with uses in smart healthcare, smart transportation, and smart cities [1]. However, the massive amounts of data in IoT impose limitations on traditional centralized machine learning in terms of network resources, data privacy, etc. The proposal of federated learning (FL) provides an effective solution to deep learning problems involving data privacy issues. Clients can collaborate with other clients in training a global model without the need to share their local data [2]. FL has been successfully applied in many domains [3,4,5,6,7]. However, the presence of data heterogeneity among clients adversely affects the model convergence and training accuracy of FL.

In real-world scenarios, the local datasets among different clients exhibit heterogeneity, indicating that their local data distribution differs from the global data distribution within the entire federated learning (FL) system. Several studies have demonstrated that the heterogeneity of data among clients significantly impacts the effectiveness of FL methods, leading to a substantial reduction in model accuracy [8,9]. Specifically, heterogeneity among clients can lead to inconsistent convergence targets for their local training. Aggregating local models with biased convergence targets will naturally result in a global model with biased convergence targets as well. Therefore, the divergence of the global model obtained from non-IID datasets as opposed to IID datasets continues to grow, which may lead to slower convergence and poorer learning performance [10]. Effectively mitigating the adverse effects of data heterogeneity on FL system models remains one of the central challenges in current federated learning optimization. This research will primarily focus on the issue of data heterogeneity in the field of horizontal federated learning.

Some researchers consider only a single category of non-IID environments and do not provide stable performance improvements in different categories of non-IID environments [11,12,13,14,15,16]. Furthermore, The authors in [17] restrain the local model update and mitigate the degree of “client drift” by introducing a variety of proximal terms. Their approach of introducing proximal terms is effective, but also inherently limits the potential for local model convergence while incurring considerable communication, computation, and storage overhead on the clients, which are intolerable for real-world distributed systems. Moreover, most previous work [11,12,13,14,15,16,17,18,19] assumes that all clients in the FL system can participate in each round of iteration. The number of clients is usually smaller when all clients participate, while in practice, the number of clients is typically larger. The scenario with a multitude of participants, where all clients participate in each FL round, is not feasible due to differences in communication or computational power, among other factors. In common approaches, the server typically employs a random selection policy to select participants and uses a model aggregation algorithm to update the weights of the global model. The random selection of clients for participation in the model aggregation process can increase the bias of the global model and exacerbate the negative effects of data heterogeneity. Therefore, it is crucial to design an optimal client selection strategy that is robust for FL.

Numerous studies focus on devising client selection strategies to alleviate the issue of data heterogeneity in FL. Some authors measure the degree of local dataset skews by utilizing the discrepancy between local and global model parameters for the development of client selection strategies [20,21,22]. These methods either rely on a global shared dataset or cause a huge waste of resources. The training loss generated during local training naturally reflects the degree of skew in the local data distribution and training progress between different clients. Other than that, The calculation and upload of the loss value will not generate new computational or storage burdens. Some studies use that biased selection based on client-side local training loss values and achieve good results [23,24,25]. They believe that favoring clients with higher local loss values can accelerate the convergence of FL in heterogeneous environments. Intuitively, in the early stages of FL, clients with high loss values will help the global model converge faster. However, choosing clients with high loss values may negatively impact accuracy improvement when the global model is close to convergence. The paper [26] points out that always selecting prioritized clients tends to result in sub-optimal performance; there is a trade-off between selecting prioritized clients and selecting a more diverse clientele. Designing an FL client selection mechanism that balances exploitation and exploration is challenging.

Deep reinforcement learning (DRL) excels at handling optimal decisions in complex dynamic environments, where the agent repeatedly observes the environment, performs actions to maximize its goals, and receives rewards from the environment. Constructing an agent for the server in FL, the agent adaptively selects clients with high or low loss values to participate in the global model aggregation process by designing a suitable reward function, thus alleviating the problem that client selection strategies are difficult to formulate in dynamic environments. We propose a DRL-based FL framework. In this framework, we design a client selection strategy in FL by utilizing the improved DDQN algorithm that makes optimal decision for the client selection problem at each iteration and selects a subset of clients to achieve the target accuracy with fewer communication rounds.

Our main contributions are as follows:

- We model the client selection problem in FL as a Markov decision process (MDP), introduce a top-p sampling strategy [27] to the double-DQN (DDQN) algorithm, and propose a new DDQN-based client sampling algorithm. In each round of iteration, this method can select a subset of clients for model aggregation, thereby significantly reducing the number of communication rounds needed for FL convergence.
- FedRLCS is deployed on the server side and does not cause additional computing or storage burdens on the client’s side. Moreover, our method represents an optimization at the system level, and it is orthogonal to other FL optimization methods.
- We summarize some classification methods and use them to construct experimental datasets with non-IID distributions. We conducted extensive experiments and the experimental results show that our method outperforms existing non-IID FL methods.

The rest of this paper is organized as follows. Section 2 summarizes related work on non-IID federated learning. We introduce the preliminaries in Section 3. We present the proposed FedRLCS in detail in Section 4. Subsequently, in Section 5, we present the experimental setup and analyze the obtained results. Section 6 presents the conclusions and future research directions.

## 2. Related Work

Federated learning trains a global model through the cooperation of multiple clients; however, data heterogeneity among different clients affects the performance of the aggregated global model. There has been a surge in research literature on the impact of reducing non-IID data on FL. We summarize strategies to overcome non-IID in existing work into the following three categories:

### 2.1. Data-Based Approaches

Several studies have attempted to alleviate the non-IID issue among clients. Zhao et al. [28] improved training on non-IID data by constructing a small, globally shared, uniformly distributed data subset for all clients. Similarly, Seo et al. [29] mitigated the quality degradation problem in FL via data sharing, using an auction approach to effectively reduce the cost, while satisfying system requirements for maximizing model quality and resource efficiency. In [30], the authors assume that a small segment of clients are willing to share their datasets, and the server collects data from these clients in a centralized manner to aid in updating the global model. Although such data-sharing-based methods have obtained significant performance improvements, they go against the original intention of FL and pose a threat to privacy. And in the absence of the client’s original data, the server cannot obtain the global data distribution information and use it to build a globally shared IID dataset.

### 2.2. Algorithm-Based Approaches

Another research aspect focuses on addressing the negative impact of heterogeneous data by designing algorithms to enhance the local training phase or improve the global aggregation process. In [11], the authors introduce a new algorithm called SCAFFOLD. The algorithm uses control variables to correct for local updates, preventing “client drift”, and leverages the similarity in client data to accelerate the convergence of FL. Li et al. [12] balances the optimization differences between global and local objectives using a regularization term. In addition, the authors [13] introduced a normalized averaging algorithm called FedNove. This algorithm normalizes local updates by the number of local training iterations per client. It ensures rapid error convergence while maintaining objective consistency. The authors of [14] propose the FedRS method, which constrains the updates of missing category weights during local training via a classification layer in a neural network. MOON [15] is proposed as model-contrastive federated learning. It introduces a contrastive loss for the clients, utilizing the representations of the global model and historical local models for learning, to correct the local model updates of each client. Similarly, the authors of [16] proposed FedProc, a prototypical contrastive federated learning approach. The authors design a global prototypical contrastive loss for local network training and use prototypes as global knowledge to correct local training for each client. The authors of [18] demonstrate a contribution-dependent weighting design, named FedAdp. It calculates the association between the client’s local goals and the global goal of the overall FL system based on the gradient information during the training process, assigning different weights to each participating client. Zhang et al. [19] address the challenge of direct model aggregation by transferring knowledge from the local model to the global model through data-free distillation. Long et al. [31] propose FedCD, which removes classifier bias from non-IID data by introducing hierarchical prototype comparison learning, global information distillation, and other methods to understand the class distribution of clients.

### 2.3. System-Based Approaches

In addition, several studies have attempted to design client selection policies for servers. In [20], the authors determine the level of IID data among clients by analyzing differences in local model weights. They assign a higher probability of selection to clients with lower degrees of non-IID, ensuring their more frequent participation in FL training. But the assumption of accessible IID public data is challenging to meet in the real world. Wu et al. [21] use the inner product of the local model gradient and the global model gradient as a measure to determine the subset of clients participating in model aggregation, ensuring that clients contributing more to reducing the global loss have a higher probability of being selected. Some studies have designed a client selection strategy by considering the local training loss values. Goetz et al. [25] evaluate the contribution of different client data in each FL round according to the local loss value, calculate the corresponding evaluation score, and select an optimized subset of clients according to the evaluation value. Cho et al. [23] theoretically demonstrate that favoring client selection with larger local loss values can improve the convergence rate compared to random client selection. Other studies employ reinforcement learning to select clients for servers. Chen et al. [32] use an UCB approach to heuristically select participating clients during each round of optimization, utilizing the cosine distance weights (CDW) of the historical global model and the current local model to measure the client’s contribution and assign rewards. Moreover, the author of [22] proposed an experience-driven control framework that uses a deep reinforcement learning algorithm to intelligently select clients in each round of federated learning (FL) by reducing the dimensionality of clients’ local model weights and using them as states to enhance the performance of the global model. Xiao et al. [33] proposed a client selection strategy based on clustering and bi-level sampling. Firstly, a subset of candidate clients is constructed using MD sampling, and then a WPCS mechanism is proposed to collect the weighted per-label mean class scores of the clients to perform clustering and select the final client.

Most of the mentioned works do not pay attention to the resource constraints of the client, which will impose a large computational or memory burden on the client. In addition, some of these works consider that the non-IID environment is single or the neural network used is relatively simple. Our work will optimize the performance of non-IID FL under the premise of protecting privacy and considering the above factors.

## 3. Preliminaries

### 3.1. Federated Learning

FL has gained recent popularity as a decentralized machine learning framework, typically consisting of a server and a set of clients. Clients in the system share locally trained model parameters rather than private local datasets. The federated averaging algorithm (FedAvg) has become the most commonly used FL algorithm. When the iteration number is *t*, each client i∈N downloads the global model parameters $w_{t}$ from the central server, and then uses the local dataset to perform SGD to optimize it and update model parameters $w_{t+1}^{i}$.

(1) $$w_{t+1}^{i}=w_{t}-\eta ▽F_{i}\left(w_{t}\right)$$

where η is the learning rate and $▽F_{i}\left(\cdot \right)$ is the gradient of client *i*. The symbols used in this paper and their definition are given in Table 1. After receiving the local model parameters $w_{t+1}^{i}$ uploaded by all clients, the FL server performs the average algorithm to update the global model parameters to $w_{t+1}$, as shown in Equation (2).

(2) $$w_{t+1}\leftarrow \sum_{i\in k}^{}\frac{1}{k}w_{t+1}^{i}$$

The above operation is repeated until the global model reaches the target accuracy.

### 3.2. Deep Reinforcement Learning

Deep reinforcement learning (DRL) is the learning process of an agent that acts by interacting with the environment to maximize the reward obtained. Specifically, at each episode *t*, the DRL agent observes the current state $s_{t}$ and chooses the action $a_{t}$ accordingly. After the action is executed, the state of the environment transitions to the next state $s_{t+1}$, and the agent obtains the reward $r_{t}$ at the same time. The DRL agent obtains and analyzes a series of traces $\left(s_{t},a_{t},r_{t},s_{t+1}\right)$ to maximize the expected cumulative discounted return $R=\sum_{t=1}^{T}\gamma^{t}r_{t}$, where γ∈(0,1) is the discount coefficient.

In the value-based DRL algorithm, the agent trains a multi-layer neural network that, for a given state vector $s_{t}$, outputs an action-value vector $Q\left(s_{t},a;\theta \right)$, where θ denotes the parameters of the network. Then, the DRL learning problem involves minimizing the MSE loss between the target and eval, which is defined as:

(3) $$L\left(\theta \right)={\left(Y_{t}-Q\left(s_{t},a;\theta \right)\right)}^{2}$$

where $Y_{t}$ is the target *Q*-value at round *t* defined as

(4) $$Y_{t}=r_{t}+\gamma \max_{a}Q\left(s_{t+1},a;\theta \right)$$

### 3.3. Effect of the Non-IID Dataset on FL Convergence

The effectiveness of FL in the IID setting was demonstrated in [2]. Nevertheless, in practical scenarios, the distribution of datasets is often non-IID, which will seriously affect the performance of the aggregated global model. The reason behind this is the heterogeneity of data, which leads to inconsistent objectives for each client executing the local SGD algorithm. In other words, for local tasks on some clients, the local model is updated toward the local optimum with the iteration of the local training. But this update path may be far from the desired optimal goal. This will cause the aggregated global model to be far from the global optimal [17]. In particular, as the FL communication rounds progress, the deviation between the global model and the global optima accumulates. This factor will lead to a converged global model with significantly lower accuracy in comparison to the scenario where the data distribution is IID.

Although existing FL methods have made considerable progress in solving non-IID data issues by limiting the degree of deviation of local model weight updates and setting a global shared dataset, the potential to suppress these disadvantages by selecting suitable participants before each communication round has been largely ignored. Performing active selection of an optimal subset of clients by comparing local training information for each client is a promising optimization method. This selection helps reduce the discrepancy between the global model parameters and the ideal model parameters, facilitating the rapid convergence of the global model toward the desired accuracy

### 3.4. Categories of Non-IID Dataset

The experiments conducted in existing studies are limited in their adoption of non-IID dataset partitioning strategies, hindering their representativeness. Therefore, we summarize several real-world common non-IID categories as the basis for our experiments and obtain a more complex and comprehensive non-IID dataset through the combination of different deviation categories.

#### 3.4.1. Quantity Bias

Quantity bias is the most common non-IID category, which means that different clients have different amounts of data [17].

#### 3.4.2. Proportional Bias

In each client’s local dataset, the proportion of data with different labels is different [22]. For example, clients from cities have more data for cats, and clients from the countryside have more data for cows. We call the label class with the highest percentage the dominant category, and the level of non-IID bias can be adjusted by the proportion of the dominant category.

#### 3.4.3. Compositional Bias

Compositional bias refers to the scenario where certain label classes are missing from a client’s dataset, meaning that training on these local datasets cannot capture the full scope of knowledge. This type of bias generally leads to a higher distribution shift [2].

#### 3.4.4. Context Bias

This concept was proposed by He et al. [34]. Data belonging to the same class from different clients have different contexts. For example, brown bears, black bears, and bears in water all belong to the same class but are distributed among different clients. This bias is closer to the real situation.

When the distribution of local datasets includes various non-IID categories, it presents a significant challenge to the convergence of FL. The synthetic dataset approach used in our experiments is detailed in Section 4.4.

## 4. Methodology

In this section, we propose a DRL-based FL framework, FedRLCS, which intelligently selects clients participating in aggregation in each round via a DRL proxy to speed up the convergence of the global model in heterogeneous environments. We introduce the overall architecture of the proposed FedRLCS in Section 4.1, detail the design of the DRL components in Section 4.2, and describe our client selection algorithm in detail in Section 4.3. Finally, Section 4.4 describes the construction method of the synthetic dataset in this paper.

### 4.1. Architecture of FedRLCS

**Figure 1:**
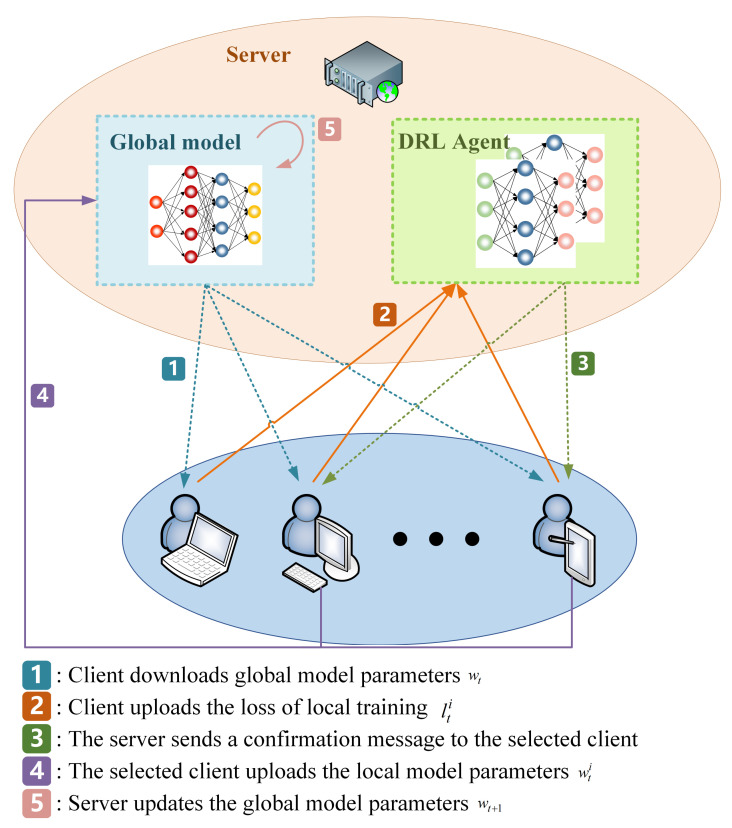
Workflow of FedRLCS.

We will now discuss a hypothetical situation in which a FL framework consists of a central server and *N* clients. In each round, *K* clients actively participate in the aggregation process of the model. Initially, the server randomly assigns weight parameters $w_{t}=w_{0}$ to the global model. Figure 1 shows the workflow of FedRLCS, following the steps below:

Step 1: The central server distributes the global model weight parameters $w_{t}$ to all *N* clients.

Step 2: Each client conducts an epoch training session using their own dataset and then submits the loss values to the server.

Step 3: The server uses the set of loss values $\left\{l_{t}^{1},l_{t}^{2},\cdots ,l_{t}^{N}\right\}$ uploaded by each client as the state $s_{t}$ of the FL in round *t*, where $l_{t}^{i}$ is the local loss value uploaded by client *i*$\left(i\in N\right)$ in round *t*. Then, the DRL agent selects *K* clients as participants in this FL round based on the state $s_{t}$ using the client selection strategy proposed in this paper and sends a confirmation message to them. The specific client selection strategy algorithm is presented in Section 4.3.

Step 4: The client receiving the confirmation message will continue the remaining local training tasks and upload the local model parameters $w_{t}^{i}$ to the server.

Step 5: After all of the selected clients have uploaded the model parameters, the server utilizes the standard FedAvg algorithm to update parameters of the global model, denoted as $w_{t+1}$.

Repeat steps 1 to 5 in a loop until the test accuracy of the global model reaches the expected goal of the FL system. For each client, no additional computation or storage overhead is introduced, and the communication overhead caused by uploading local training loss values is almost negligible. In addition, clients not selected in Step 3 do not need to complete local training tasks, which minimizes unnecessary resource consumption. Algorithm 1 shows the framework of FedRLCS in detail.
**Algorithm 1** FedRLCS Algorithm1:**procedure** Client-Selected Federated Learning2:    Server initialization global model parameters $w_{t}=w_{0}$;3:    **for** t=1,2,⋯ **do**4:        push $w_{t}$ to each client;    **//Step 1**5:        **for** each client i∈N in parallel **do**6:           $l_{t}^{i}\leftarrow$ ClientCheck($i,w_{t}$);    **//Step 2**7:        **end for**8:        $s_{t}\leftarrow \left\{l_{t}^{1},l_{t}^{2},\cdots ,l_{t}^{N}\right\}$;9:        $a_{t}\leftarrow$ Client Selection strategy($s_{t}$);    **//Step 3**10:       **for** each client $i\in a_{t}$ in parallel **do**11:            $w_{t+1}^{i}\leftarrow$ClientUpdate(*i*);    **//Step 4**12:       **end for**13:        $w_{t+1}\leftarrow \frac{1}{K}\sum_{i\in a_{t}}w_{t+1}^{i}$;    **//Step 5**14:    **end for**15:**end procedure**16:   17:**function** ClientCheck($i,w_{t}$)18:    $w_{t}^{i}\leftarrow w_{t}$;19:    **for** local training epoch e=1 **do**20:         $w_{t}^{i}\leftarrow w_{t}^{i}-\eta ▽F_{i}\left(w_{t}^{i}\right)$;21:    **end for**22:    return training loss value $l_{t}^{i}$;23:**end function**24:   25:**function** ClientUpdate(*i*)26:    **for** local training epoch *e* from 1 to E−1 **do**27:         $w_{t+1}^{i}\leftarrow w_{t}^{i}-\eta ▽F_{i}\left(w_{t}^{i}\right)$;28:    **end for**29:    return $w_{t+1}^{i}$;30:**end function**

### 4.2. Design of DRL Model

We transform the client selection problem in FL into a DRL paradigm and provide a comprehensive explanation of the underlying definitions.

#### 4.2.1. State Space

The state of the FL system in each round is closely related to the local training process of each client, and the local models provided by any client have a significant impact on the aggregation of the global model. The server should make optimal decisions based on the training states of all clients. The training loss is generated by clients during local training and naturally reflects the level of optimization of the global model on the client side. It implicitly reveals the interconnection between different clients. Therefore, we regard each FL round as a step of DRL, and the state $s_{t}$ of the *t*-th step of DRL is the set of local training loss values of all clients in the *t*-th FL round; that is, $s_{t}=\left\{l_{t}^{1},\cdots ,l_{t}^{N}\right\}$.

In the state vector collection phase, we do not require all clients to complete a full local training task; only the loss value calculated from one gradient computation round is needed as the state vector. Compared to the dimensionality reduction encoding of local model parameters as the state, as proposed by Wang et al. [22], our method offers an advantage in that updating loss values incurs minimal additional computational and communication costs, making it better suited for real-world applications. Furthermore, loss values can also serve as valid status inputs.

#### 4.2.2. Action Space

In each FL round, the server selects *K* participants from all *N* clients; therefore, the action of each round *t* is defined as $a_{t}=\left\{i_{1}\cdots i_{K}\right\}$, where $i_{K}$ is the client number.

#### 4.2.3. Reward Function

The proper design of the reward function is crucial for ensuring the effectiveness of the DRL model, and it should be aligned with the objective of optimization. Our goal is to be able to make the global model converge on the global target with less communication cost in the non-IID environment. The higher the accuracy achieved by the global model test, the higher the reward for the agent. In addition, as the improvement in model accuracy becomes more challenging during later iterations compared to earlier iterations, higher rewards should be given during the later iterations. So our reward function is defined as:

(5) $$r_{t}=\psi^{\delta_{t}-\delta }-1$$

where ψ represents a constant term, $\delta_{t}$ refers to the accuracy of the global model in round t, and δ is the target accuracy of FL.

### 4.3. Client Selection Strategy

In this paper, we improve the DDQN algorithm [35] to train the client selection policy in the FL system. DDQN consists of two neural networks. The eval network is utilized to establish the greedy policy, while the target network is employed to ascertain its corresponding value. In addition, the agent maintains an experience replay buffer *M*, allowing for the repeated learning of old experiences to increase sample utilization. Algorithm 2 shows the client selection strategy and the DRL agent-training process.
**Algorithm 2** Client selection algorithm based on DDQN.1:**procedure** Agent training2:    Initialization θ, $\theta^{-}$, *M*;3:    $\theta^{-}=\theta$;4:    **for** each communication round t=1,2,⋯ **do**5:        Feed the state $s_{t}$ to the eval network to obtain the *Q*6:        value;7:        Use the top-p sampling strategy for *Q* to obtain8:        action $a_{t}$;9:        Obtain reward $r_{t}$ according to Equation (5);10:        Obtain new state $s_{t+1}$;11:        store experience tuple $\langle s_{t},a_{t},r_{t},s_{t+1}\rangle$ in *M*;12:        **for** episode j=1 to *J* **do**13:           Randomly sample a mini-batch of experiences14:           from *M*;15:           $Y_{t}=r_{t}+\gamma Q\left(s_{t},\underset{a}{\arg\max}Q\left(s_{t},a;\theta_{t}\right);\theta_{t}^{-}\right)$;16:           $L\left(\theta_{t}\right)={\left(Y_{t}-Q\left(s_{t},a;\theta_{t}\right)\right)}^{2}$;17:           Minimize $L\left(\theta_{t}\right)$ and update weights $\theta_{t}$;18:           Every τ steps, reset $\theta_{t}^{-}$ = $\theta_{t}$;19:        **end for**20:    **end for**21:**end procedure**

The agent randomly initializes the eval network parameters θ, the target network parameters $\theta^{-}$, and the experience replay buffer *M*. In each round of communication, the agent feeds the state vector $s_{t}$ to the eval network and computes the action-value vector $Q(s_{t},a;\theta )$. The agent selects a set of clients corresponding to the action ($a_{t}$) to participate in this FL round by the top-p sampling strategy. First, the action-value vector $Q(s_{t},a;\theta )$ is normalized to obtain a distribution $P\left(x\right)$ and construct a minimum candidate subset $N^{\left(p\right)}\subset N$, such that

(6) $$\sum_{x\in N^{\left(p\right)}}P\left(x\right)\geq p$$

where *p* is the probability threshold, which represents the proportion of the probability density of the candidate subset in the entire probability distribution. Rescale the distribution $P\left(x\right)$ to $P^{\prime }\left(x\right)$:

(7) $$P^{\prime }\left(x\right)=\left\{\begin{array}{cc} P\left(x\right)/\sum_{x\in N^{\left(p\right)}}P\left(x\right)\; & \;\mathrm{if}\;x\in N^{\left(p\right)}\\ 0\; & \;otherwise. \end{array}\right.$$

Action $a_{t}$ is sampled based on the new distribution $P^{\prime }\left(x\right)$. Compared with the greedy sampling strategy in the DDQN algorithm, the top-p sampling strategy is more suitable for FL tasks. This strategy can better prevent the agent’s neural network from falling into local optima and prevent the agent from selecting only clients with high-quality local model parameters.

All clients that receive the confirmation message will complete the remaining local training and upload the trained local model parameters to the server. After the global model is aggregated and updated, the agent calculates the reward $r_{t}$ with Equation (5), enters the next moment $s_{t+1}$, and stores the experience tuple $\langle s_{t},a_{t},r_{t},s_{t+1}\rangle$ into *M*. During the agent-training phase, a mini-batch of experience samples is randomly sampled from *M* at each iteration to calculate the loss function $L_{\theta }$, which is the same as Equation (3). The target value $Y_{t}$ is defined as:

(8) $$Y_{t}=r_{t}+\gamma Q\left(s_{t+1},\underset{a}{\arg\max}Q\left(s_{t+1},a;\theta_{t}\right);\theta_{t}^{-}\right)$$

where γ is the discount factor. θ denotes the eval network parameters, and $\theta^{-}$ denotes the target network parameters. The eval network parameters are then updated using gradient descent.

(9) $$\theta_{t+1}\leftarrow \theta_{t}+\lambda \left(Y_{t}-Q\left(s_{t},a;\theta_{t}\right)\right){▽}_{\theta_{t}}Q\left(s_{t},a;\theta_{t}\right)$$

In every τ step, the model parameters of the eval network are copied to the target network.

The neural network of the DRL agent only requires two hidden layers, and DRL training is performed on a powerful central server. Therefore, the time cost incurred by training the agent’s decisions in the federated learning process can be almost negligible.

### 4.4. Synthetic Dataset Approach

We design different synthetic datasets based on the non-IID data categories introduced in Section 3.4 to simulate real data heterogeneity scenarios. Table 2 summarizes the characteristics of different synthetic datasets.

#### 4.4.1. Dominant Class Partition

**Figure 2:**
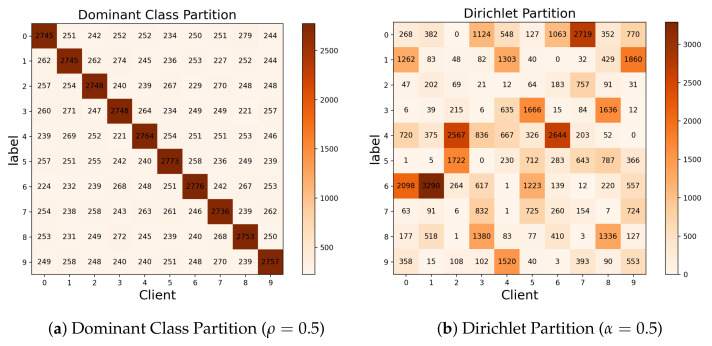
Visualization of the CIFAR-10 dataset under different partitioning strategies.

Client data consist of a dominant class and other classes, with the majority belonging to the dominant class. We use ρ to represent the proportion of the dominant class, for example, ρ = 0.8 means that 80% of the data belong to the same class. The other 20% of the data consist of random samples from other classes of data, where a higher ρ indicates higher data heterogeneity. The amount of data is basically the same between different clients. The contexts of different clients with the same dominant class are different for the NICO dataset. It means that even if different clients have the same label, context bias is still a problem. Figure 2a shows the visualization after partitioning the CIFAR-10 datasets to 10 clients using the dominant class partition strategy.

#### 4.4.2. Label-Based Dirichlet Partition

This partitioning strategy has recently been applied in the construction of non-IID datasets in many studies [8,36]. Non-IID datasets between different clients are generated by using the Dirichlet distribution Dir(α), with a lower concentration parameter α representing a higher degree of non-IID. The distribution of data volume and labels among different clients is unbalanced. Figure 2b shows the visualization after partitioning the CIFAR-10 datasets to 10 clients using the Dirichlet partition strategy.

## 5. Experiments and Results

### 5.1. Experimental Setup

We compare FedRLCS with five state-of-the-art approaches: FedAvg [2], FedProx [12], SCAFFOLD [11], MOON [15] and FedDyn [37]. These strategies were proposed as improvements to address the issue of data heterogeneity in FL. Comparing our proposed method with these approaches can highlight the superiority of our method. We conduct experiments with different network architectures on four real datasets, respectively.

For the CIFAR-10 dataset, we use the classic LeNet-5 as the base encoder. The number of clients (N) is set to 100. For each client, the batch size is set to 50, and the number of local epochs is set to 5.

For the CIFAR100 dataset. we train it using a lightweight network MobileNetV2. The number of clients (N) is 50. The batch size and local epochs are the same as in the CIFAR10 dataset.

The NICO dataset was constructed and published by He et al. [34]; it consists of two superclasses (vehicle and animal) for non-IID image classification problems. We use the animal superclass as our dataset, which consists of 10 classes with a total of 10 contexts for each class. The number of images is 12,811, and we divide the training set and test set at a ratio of 8:2. We train it with ResNet-18. The number of clients (N) is 50, the batch size for each client is 16, and the local epoch number is 5.

For the Tiny ImageNet dataset, we train it using the ResNet-34 network. The total number of clients, *N*, is 100. Each client has a batch size of 16, and the local epoch number for each client is set to 5.

We utilize the Adam optimizer with a learning rate of 0.001 for all methods. In each round of communication, by default, 10 clients are selected as participants from all clients. For FedProx, the proximal term μ = 0.01. For MOON, the temperature parameter is set to 0.5, and the model-contrastive loss weight parameter $\mu_{MOON}$ = 1. In addition, an additional projection head composed of a 2-layer MLP is added to the fixed network structure, and the output size is 256, which is the same as in the paper [36], while other methods use the original network architecture.

The DRL agent’s model includes two four-layer MLP networks, each with an input size of *N* and an output size of *K*. The hidden layer sizes are 256 and 128, respectively. The probability threshold *p* of the top-p sampling strategy is set to 0.9. We use Adam as the optimizer of the agent neural network with a learning rate of 0.01.

### 5.2. Communication Efficiency

We calculate the test accuracy when the standard FedAvg algorithm almost converges in each heterogeneous environment, as our target accuracy. Using the aforementioned default settings, the communication iteration number needed for various methods to attain the target accuracy is shown in Table 3, where ‘N/A’ means that the method failed to achieve the target test accuracy within 1000 epochs.

**Figure 3:**
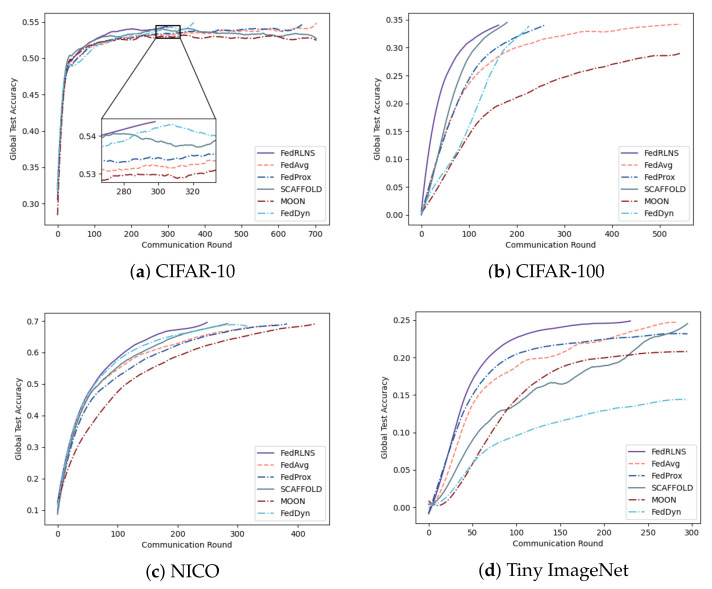
The test accuracy of each communication round of different FL methods on the non-IID dataset constructed by the dominant class partition strategy, where ρ=0.5.

**Figure 4:**
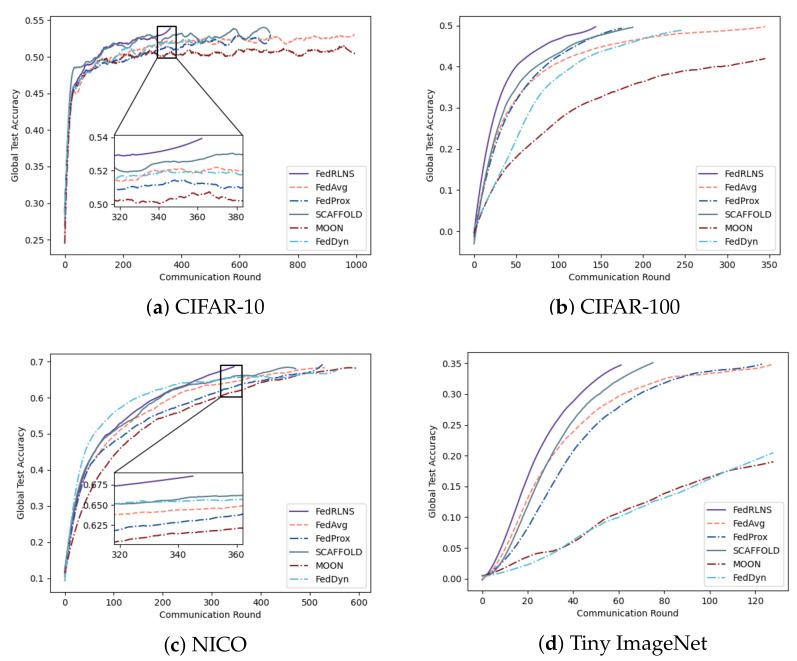
The test accuracy of each communication round of different FL methods on the non-IID dataset constructed by the Dirichlet partition strategy, where α=0.5.

Figure 3 and Figure 4 show the process of the test accuracy of the global model, trained by different FL methods, changing with the communication rounds under the two partition strategies, respectively. Comparing different FL methods, we observe that our method (FedRLCS) is more effective than other FL methods in both partition strategies across different datasets. Specifically, on the CIFAR-10 dataset, our method (FedRLCS) requires 58% and 64% fewer communication rounds to achieve the target accuracy than the baseline FL method FedAvg. On the CIFAR-100 dataset, FedRLCS needs only 165 and 145 communication rounds to attain the target accuracy, reducing the number of communication rounds by 70% and 58% compared with FedAvg. On the NICO dataset, FedRLCS requires 250 and 346 communication rounds to achieve 70% accuracy, which is 32∼35% fewer than the FedAvg method and 12∼27% fewer than the suboptimal FL method SCAFFOLD. On the Tiny ImageNet dataset, which has multiple label quantities, our method converges to the target accuracy in only 81% and 48% of the communication rounds required by the FedAvg method. Other methods perform even worse than FedAvg.

Due to the lack of optimization for the non-IID environment, FedAvg usually needs to iterate more communication rounds. The effect of the MOON method is much worse than other methods; even the target accuracy cannot be achieved because the extra projection head will affect the performance of the original network architecture. The performance of the FedDyn method is second only to FedRLCS on the CIFAR-10 dataset with a simple network architecture; however, it exhibits unsatisfactory performance on the CIFAR-100, NICO, and Tiny ImageNet datasets, which have more complex network architectures. FedRLCS achieves optimal performance on the non-IID dataset with different partitioning strategies, demonstrating the effectiveness of our proposed DDQN-based client selection strategy.

### 5.3. Heterogeneity

We examine the impacts of different data heterogeneity levels by altering the concentration parameter α of the Dirichlet partition and the proportion parameter ρ of the dominant class partition. This ensures that our proposed method can be adapted to different environments. Higher data heterogeneity is indicated for smaller α and larger ρ, respectively. The results in Table 4 show that FedRLCS consistently maintains the best performance in environments with different degrees of heterogeneity.

**Figure 5:**
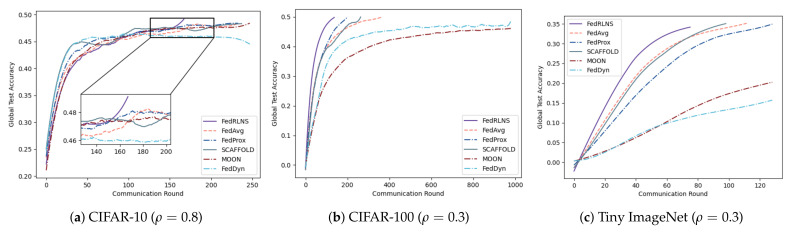
Test accuracies for different degrees of heterogeneity under the dominant class partition.

**Figure 6:**
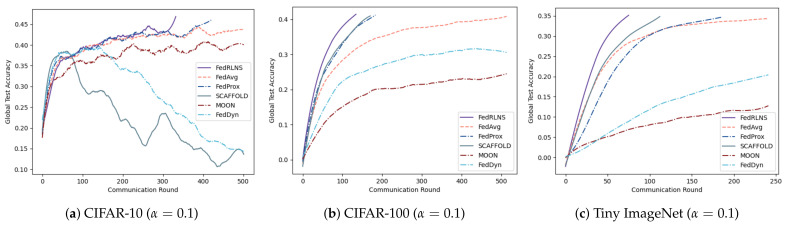
Test accuracies for different degrees of heterogeneity under the Dirichlet partition.

Compared with the FedAvg method, as the data heterogeneity changes, FedRLCS reduces communication rounds by 28% and 34% on CIFAR-10 (ρ=0.8, α=0.1), by 62% and 78% on CIFAR-100 (ρ=0.3, α=0.1), and by 33% and 69% on Tiny ImageNet (ρ=0.3, α=0.1). We should note that when the data are highly heterogeneous, the performance of FedDyn will decrease and cannot converge to the target accuracy. SCAFFOLD and MOON fail to achieve target accuracy on CIFAR-10 (α=0.1). The accuracy change curves of the three datasets are shown in Figure 5 and Figure 6, respectively. As expected, FedRLCS is effective and robust in FLs with different degrees of heterogeneity; this is because the DRL agent can adaptively select the appropriate participants to aggregate the global model based on the environment, reducing the target skews of the global model.

### 5.4. Scalability

**Figure 7:**
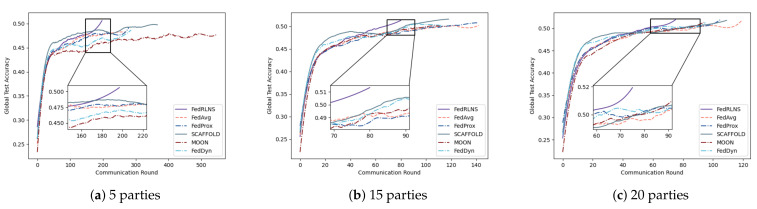
Test accuracies for different numbers of participants $\left\{5,15,20\right\}$ on the CIFAR-10.

To demonstrate the scalability of FedRLCS, we construct two different setups. The comparison between FedRLCS and other FL methods is shown in Table 5:

Setting 1: We experimented with varying numbers of parties on CIFAR-10 (α=0.5) to observe its effect on FL convergence. Specifically, with a total number of clients, N=100, the number *K* of clients participating in the aggregation changed to 5, 15, and 20. The results are shown in Figure 7. In comparison to the FedAvg approach, FedRLCS reduced communication rounds by 26∼43%. Surprisingly, it was observed that when the number of participants in the FL system decreased to 5, other non-IID FL methods performed even worse than FedAvg. When only a few clients participate in FL and cannot provide high-quality updates to the global model, FL methods that rely on the experience of the global model to aid local training perform unsatisfactorily. FedRLCS, which considers the degree of local training for each client and performs selective aggregation, still maintains good performance.

**Figure 8:**
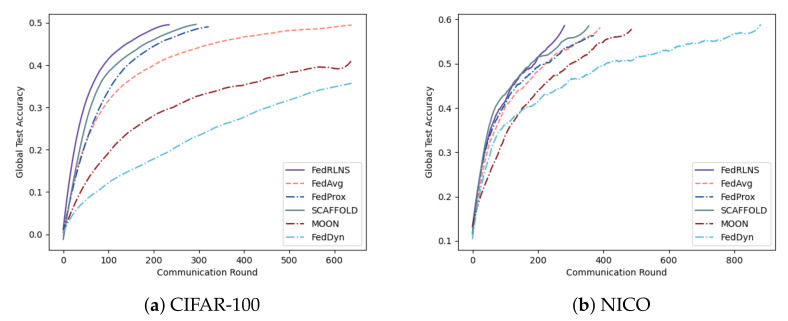
Test accuracies of CIFAR-100 and NICO when the total number of clients N=100.

Setting 2: We expand the total number of clients *N* on CIFAR-100 (α=0.5) and NICO (α=0.5) to 100, and the *K* number remains unchanged. The results are shown in Figure 8. Despite the increased space for selection, FedRLCS ensures that the global model converges first, maintaining a substantial advantage over other non-IID FL methods.

## 6. Conclusions

This paper introduces a DRL-based FL framework to address the data heterogeneity challenge in FL. Specifically, we use the improved DDQN algorithm to train an agent in the server, which can actively select specific clients to participate in the aggregation process of the global model in each FL round communication, preventing bias in model updates caused by certain skewed local models. Extensive experimental results indicate that compared to other FL optimization algorithms targeting data heterogeneity, FedRLCS reduces the number of epochs by 20∼64% on CIFAR-10, 10∼70% on CIFAR-100, 12∼35% on NICO, and 18∼52% on Tiny ImageNet. Existing methods do not show significant advantages (or even disadvantages) over FedAvg in all heterogeneous environments. In contrast, FedRLCS has demonstrated its strengths in different experimental setups. FedRLCS is a systems-based optimization approach that is compatible with previous work. In addition, the training of the agent network is deployed on the server, without introducing an additional workload for the client. Our proposed approach can be better applied in resource-constrained FL environments. One limitation of our study is the exclusive focus on image tasks. The application of Federated Learning (FL) in natural language processing and graph neural networks is extensive and warrants further exploration. In addition, future work will consider factors such as model accuracy, local computation, and communication times. This approach will be especially relevant for more complex problems, such as those involving environmental loads on road transport networks.

## Figures and Tables

**Table 1:** The definition of the symbol used in this paper.

Symbol	Definition
*N*	total number of clients
*K*	The number of clients selected in each FL round
$w_{t}$	Global model parameters for round *t* of FL
$w_{t}^{i}$	Local model parameters of client *i* in FL round *t*
η	Learning rate for local training
$s_{t}$	State of FL in round *t*
$a_{t}$	Subset of clients selected by FL in round *t*
$r_{t}$	Reward for round *t*
γ	discount coefficient
$Y_{t}$	The target Q value of round *t*
ψ	Constant term
δ	Target Accuracy of FL
$\delta_{t}$	Test accuracy for round *t* in FL
θ	Parameters of the eval network
$\theta^{-}$	Parameters of the target network
$L\left(\cdot \right)$	Agent loss function
$Q\left(\cdot \right)$	Action-value of the output of the agent neural network
*p*	probability threshold for top-p sampling strategy
*R*	Accumulated rewards
λ	Scalar step size
τ	The parameter update interval of the target network
*M*	experience replay buffer

**Table 2:** Characteristics of non-IID synthetic datasets. Among them, ρ represents the dominant class partition, and α represents the Dirichlet partition.

Dataset		Quantity Bias	Proportional Bias	Compositional Bias	Context Bias
CIFAR10	ρ	×	✓	×	×
✓	α	×	✓	✓	×
CIFAR100	ρ	×	✓	×	×
✓	α	×	✓	✓	×
Tiny ImageNet	ρ	×	✓	×	×
✓	α	×	✓	✓	×
NICO	ρ	✓	✓	×	✓
✓	α	×	✓	✓	✓

**Table 3:** Communication rounds required for FedRLCS and other baselines to achieve the target accuracy on synthetic datasets. (The symbol ‘N/A’ indicates that the method was unable to achieve the target test accuracy within 1000 epochs).

	CIFAR-10		CIFAR-100		NICO		Tiny ImageNet	
	ρ=0.5	α=0.5	ρ=0.5	α=0.5	ρ=0.5	α=0.5	ρ=0.5	α=0.5
acc	55%	55%	35%	50%	70%	70%	25%	35%
FedAvg	704	995	544	346	365	532	284	129
FedProx	665	707	261	176	383	527	N/A	124
SCAFFOLD	N/A	704	181	189	284	472	296	76
MOON	N/A	N/A	N/A	N/A	432	595	N/A	N/A
FedDyn	371	507	227	248	317	557	N/A	N/A
FedRLCS	299	363	165	145	250	346	231	62

**Table 4:** Number of communication rounds required for different FL methods to achieve target accuracy on CIFAR-10 (ρ=0.8, α=0.1), CIFAR-100 (ρ=0.3, α=0.1), and Tiny ImageNet (ρ=0.3, α=0.1).

	CIFAR-10		CIFAR-100		Tiny ImageNet	
	ρ=0.8	α=0.1	ρ=0.3	α=0.1	ρ=0.3	α=0.1
acc	50%	50%	50%	43%	35%	35%
FedAvg	234	502	367	668	113	242
FedProx	234	421	202	196	129	187
SCAFFOLD	238	N/A	264	191	99	113
MOON	248	N/A	N/A	N/A	N/A	N/A
FedDyn	N/A	N/A	981	N/A	N/A	N/A
FedRLCS	168	333	138	135	76	76

**Table 5:** Setting 1: On the CIFAR-10, each FL round has 5, 15, and 20 participants, respectively, with the number of epochs needed to achieve the target accuracy. Setting 2: The total number of clients N=100, and epochs needed to achieve target accuracy on CIFAR-100 and NICO.

	Setting 1			Setting 2	
	**5 Parties**	**15 Parties**	**20 Parties**	**CIFAR-100**	**NICO**
acc	52%	52%	52%	50%	60%
FedAvg	270	143	120	639	391
FedProx	280	142	106	323	372
SCAFFOLD	367	119	110	295	356
MOON	546	112	95	N/A	487
FedDyn	287	113	96	N/A	882
FedRLCS	198	81	76	235	282

## Data Availability

Data are contained within the article.
